# Kajava Classification: The Person and the Research

**DOI:** 10.1007/s00266-023-03451-7

**Published:** 2023-06-22

**Authors:** Elena Surcel, Virve Koljonen

**Affiliations:** Department of Plastic surgery, Helsinki University and Helsinki University Hospital, Park Hospital (HUS), P.O. Box 281, 00029 Helsinki, Finland

**Keywords:** Ectopic breast, Supernumerary nipples, Kajava classification

## Abstract

The Kajava classification for ectopic breast tissue is still widely used, although it was published in 1915 in Finnish. This historical note sheds light on the person and research behind the classification.

*Level of Evidence V* This journal requires that authors assign a level of evidence to each article. For a full description of these Evidence-Based Medicine ratings, please refer to the Table of Contents or the online Instructions to Authors www.springer.com/00266.

## Introduction

Ectopic breast tissue is a comprehensive term encompassing both supernumerary breasts and aberrant breast tissue. Already in 1915, Professor Yrjö Kajava authored a descriptive case series of aberrant breast tissue and supernumerary nipples in a Finnish cohort, thereby contributing to the systemic understanding of this phenomenon [1]. The classification is based on the evaluation of anatomical components, the presence or absence of areola, glandular tissue, and nipple (Table 1). The classification, which has come to be known as the *Kajava classification*, continues to hold significant relevance and widespread usage in contemporary studies [2, 3] and clinical practice [4–6]. Hence, it appears appropriate to illustrate the individual behind the eponymous classification and research culminating in its development.

**Table 1:** Kajava classification of extramammary breast tissue

Class I	*Polymastia*	Complete breast(s) with nipple, areola, and glandular tissue
Class II	*Supernumerary breast without areola*	Nipple and glandular tissue, no areola
Class III	*Supernumerary breast without nipple*	Areola and glandular tissue, no nipple
Class IV	*Mamma aberrata*	Only glandular tissue
Class V	*Pseudomamma*	Nipple and areola, no glandular tissue, replaced by fat
Class VI	*Polythelia*	Nipple only
Class VII	*Polythelia areolaris*	Areola only
Class VIII	*Polythelia pilosa*	Patch of hair only

Professor Kajava (1884–1929) was a Finnish physician and held the esteemed position of Professor of Anatomy at the University of Helsinki during the period spanning from 1921 to 1929 [7]. He had a special interest in anthropology, and he focused his scholarly efforts on the special features in Finnish people. Professor Kajava is also the pioneering author of the first Finnish-language textbook on human anatomy: *Human anatomy: a textbook for gymnastics teachers, physical therapists, and masseurs.* This seminal work had a pivotal role in introducing a multitude of new anatomical terms in the Finnish language.

The aim of the study is given in the first sentence of the article: “The presence of supernumerary mamma in humans so far seems to be a very fragmented and incompletely studied area, despite the fact that the formations mentioned are among the few atavistic rudiments which, of course, are under our study and which are relatively easy—at least in typical cases—to catch our eye.” For his study on Supernumerary nipples in Finnish people [1], Professor Kajava employed a convenience cohort. The cohort was originally used to investigate the occurrence of tuberculosis during the summer of 1914 at the Kymi Factory in Finland. This cohort comprised a total of 8246 individuals, with 4244 (51%) males. The selection of this cohort for breast anomalies was based on its suitability for studying supernumerary nipples, as the individuals were “exposed to the waist.” It is worth noting that the population of Finland in 1914 was only 3,069,500 [8], making the cohort account for a 0.3% of the total population. With the current population to achieve similar sample size would require over 16,000 persons.

Professor Kajava meticulously tabulated every case, and the parameters collected were age, gender, normal nipple laterality and diameter, and the same parameters for the supernumerary nipples and distance between the normal and supernumerary nipple.

There were altogether 142 cases, with 102 (72%) males. The over all prevalence in this cohort was thus 1.72%, with prevalence in men 2.4% (102) and 0.99% (40) in women. In the majority of the cases, 119 (62.3%), the ectopic breast was located just under the normal nipple above the lowest rib.

We extracted from the remark column seven sets of families: three brothers who all had unilateral hyperthelia and hypermastia, of note their parents did not have this anomaly, two sets of siblings with unilateral and bilateral hyperthelia and hypermastia, two sets of father and son with unilateral and bilateral hyperthelia and hypermastia, mother and daughter with unilateral hyperthelia and hypermastia, and sisters with unilateral hyperthelia and hypermastia findings. The genetics of supernumerary nipples is still pending. However, familial supernumerary nipples is a known entity, with the first family reported in modern medical literature in 1988 [9].

Kajava classification is based on meticulous listing and tabulation, with a relatively large cohort. It is remarkable that the Kajava classification remains recognized and utilized despite it being originally published more than a century ago in Finnish. This highlights its impact on medical literature, despite linguistic and temporal barriers. Or the situation described by Kajava still persist, lack of research in this subject.
